# Midwifery Care Studies: A Reflexive Methodology for a Practice‐Based Science

**DOI:** 10.1111/birt.70058

**Published:** 2026-02-05

**Authors:** Annekatrin Skeide

**Affiliations:** ^1^ Charité – Universitätsmedizin Berlin Institute of Midwifery Berlin Germany

## Abstract

**Background:**

Midwifery research is increasingly understood as shaped by the specific social, political, and historical contexts, with scientific practices actively co‐producing realities. This recently developed perspective highlights the need for discipline‐specific approaches that reflect the diversity and creativity of midwifery care.

**Aim:**

This contribution introduces Midwifery Care Studies as a novel, reflexive approach to studying midwifery as a practice‐based science.

**Findings:**

Building on analyses inspired by feminist science studies, Midwifery Care Studies examine midwifery care in practice. Using participatory methods, this approach aimed to investigate midwifery care practices on their own terms.

**Discussion:**

Midwifery Care Studies share the sensitivities and response‐abilities that shape everyday midwifery care practices. Acknowledging the relational character of midwifery care, Midwifery Care Studies articulate modes and techniques of becoming‐with that involve birth givers, fetuses, midwives, technologies, words, values, and birthing environments. By carving out the material, social, and ethical specificities of situated midwifery care practices, Midwifery Care Studies examine unfolding and shifting “goods” and “bads” in practice, as well as how tensions between them are handled.

**Conclusion:**

Sensitive to the ontological politics of care and research practices, Midwifery Care Studies aim at providing the analytical and conceptual resources needed to foster generative engagements with the multitude of lived realities of being pregnant and giving birth.

## Graphical abstract

Introducing Midwifery Care Studies: a novel reflexive approach examining midwifery as practice‐based science.

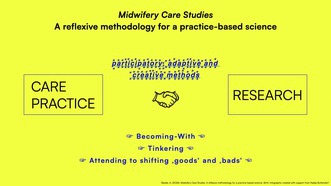

## 1 Introduction: Pressing Concerns as Matters of Care

Recently, important and pressing concerns have been raised regarding midwifery research: The “quantification of midwifery knowledge” may risk reproducing “patriarchal, colonizing and medically dominant systems of thought and knowledge creation” [1]. In order to bring about “genuine change,” midwifery and maternity care researchers are called “to hold space for qualitative expertise; for deep, slow, reflective, theoretical thinking; for exploring tacit and experiential knowledge; for tangential asides; for creativity; for meandering down various paths; for seeing what is possible; and for discussion of why these are important to midwifery research, just as we discuss how such things are important to midwifery practice” [1]. In the same vein, a call for “critical midwifery studies” has been issued, introduced as an alternative approach to “[m]ainstream midwifery research, education, practice, policy, and regulations,” which is “largely White and Western‐centric, using positivistic and universalist principles of biomedical research” [2]. Drawing on critical theory helps midwifery scholars to understand and to challenge “the existing and continuous reproduction of injustice within SRMN [sexual, reproductive, maternal and newborn] care,” the Critical Midwifery Collective Writing Group suggests [2].

Our colleagues emphasize that midwifery researchers do, sense, and think from positions that are materially, socially, politically, geographically, and historically specific [3]. They suggest that our methods and concepts contribute to shape our objects of research. Instead of merely *representing* a reality that is out there waiting to be discovered, scientific interventions *generate* certain realities. They concede that scientific practices are ordering and materially productive practices: they intervene in and co‐produce lived realities, necessarily privileging certain realities while backgrounding others. Refuting generalizing or even universalizing accounts, they think it is high time to attend to the realities that are marginalized. We are thus invited to (re)consider how we carry out midwifery research, for the questions we ask and for the answers we craft. We are asked to take responsibility by thoroughly pondering the realities that our research helps to foreground and strengthen. Our task is to systematically and strategically engage with carving out a discipline‐specific body of midwifery knowledge and analytical tools that reflect the creativity and the diversity of midwifery care practices appropriately.

Joining these calls for a reconsideration of the politics of midwifery research and care, I introduce a methodological strategy that privileges care‐specific modes of theoretical and methodical interventions in and through midwifery research in this discussion paper. Drawing on findings and strategies from feminist science and technology studies' (STS) engagements with care practices [3, 4, 5, 6], this approach, which I call Midwifery Care Studies, investigates specific and local midwifery care practices with the aim of improving them *on* and *in their own terms* [7, 8]. Avoiding to craft universalist accounts of midwifery care realities, Midwifery Care Studies engage with situated care practices, focusing on the particularities of midwifery care practices by addressing their various and shifting heterogeneous participants, values and goals. The situated insights can then be used to compare midwifery care situations, “contrasting” their differences and tensions instead of assimilating them into a “coherent narrative” or “grand total” [9].

This methodological strategy takes seriously the situated materialities, normativities, and responsibilities of both care and research practices, thereby shaping midwifery research into resonant, reflective extensions, and theoretical condensations of midwifery care practices. It privileges the articulation of messy, not necessarily coherent practical realities of midwifery care constituted of actions and effects that are “finite and dirty” instead of “transcendent and clean” [10]. Differently from approaches that conceive of midwifery care as a rational application of professional knowledge to specific situations, Midwifery Care Studies conceive of midwifery care as sets of activities that are rather open‐ended, tentative, and unfolding.

Aiming at strengthening and improving midwifery care, Midwifery Care Studies follow the Critical Midwifery Collective Writing Group's [2] strategy to engage in “contexting” midwifery care. But this approach does so by paying attention to how social positions and relationships are enacted in concrete and collective social and material interactions with institutions, “systems” and “society,” rather than taking them as explanatory macrostructures [11]. The aim is to investigate “how the social emerges, what form it takes and what collectives it creates” [12]. Interested in “hopeful analysis,” Midwifery Care Studies “are moved by situated normativities” [4], by local ways of doing “good,” by how midwives and women live up to their values and co‐shape them in practice while acknowledging that “goods” and “bads” often emerge together. Intervening medically during a physiological birth (e.g., administering oxytocin to speed up labor) can help to prevent complications, but it can leave the birthing person feeling disempowered. Supporting a birth giver's refusal of a cesarean section despite medical advice can support their autonomy but may increase risks for the fetus. Incorporating culturally specific postpartum care practices can be a strategy to provide respectful maternity care but may clash with evidence‐based recommendations and institutional policies.

Midwifery Care Studies consider that midwifery care practices are also ethical practices and as such situated in and enmeshed with specific social and material contexts. It recognizes that within the same situation, different “goods” may emerge that can clash with one another. In addition, what may be considered “good” in one situation might not hold the same value in another, highlighting the complexity of navigating values in midwifery care. By focusing on situated practices, Midwifery Care Studies aim to empirically illuminate the dynamic and relational nature of ethics‐in‐practice.

Avoiding a detached critical stance from which to pass judgment, Midwifery Care Studies analyze and compare carefully which “goods” and “bads” come to matter in midwifery care relationships and how exactly. This approach acknowledges midwifery scholars’ inextricable involvement in shaping the midwifery care practices they study and write about. By cultivating involvement with, interest in, and responsiveness to midwifery care practices, Midwifery Care Studies shed light on the creativity and generativity of these practices.

## 2 Where We Stand I: Shedding Light on Midwifery's Own Terms

In conducting clinical research and systematic reviews, midwifery researchers have strategically appropriated the evidence‐based approach by asking different questions than obstetricians. As a consequence, they have produced a strong corpus of gold‐standard evidence supporting midwifery continuity of care and its contribution to reducing perinatal morbidity and mortality worldwide, while also being positively experienced by pregnant women and people, birth givers, and parents [13, 14, 15, 16, 17, 18]. Based on this body of research, midwifery scholars have drawn up clinical guidelines and policy papers. This work has successfully enabled a position from which to negotiate with medical colleagues, policy makers, or politicians based on a partnership of equals. Nevertheless, midwife‐led, woman‐centered care models aimed at the continual attendance by midwives throughout pregnancy, labor, and postpartum have not become the global gold standard of maternal health care. Furthermore, this strategy comes at a price, our colleagues argue: the specificities of midwifery care have not been addressed *in their own terms* [1, 2].

But what are midwifery's own terms? Various suggestions have been made: midwifery scholars have, for example, stressed the importance of woman‐centered care [19]. They have described midwifery care in terms of empowerment or humanization [20, 21], emphasized the normality and physiology of birth [22], introduced alternative understandings of health, such as salutogenesis [23], and called for a care ethical approach to midwifery care [24].

However, midwifery scholars have also pointed to intricacies of midwifery care practices that are yet insufficiently articulated in midwifery research, including what exactly the position of a woman who is (together) with, as the English term midwife denotes, consists of *in practice*. In political and scientific midwifery discourses, being‐with has been framed as a strategy to promote “physiologically normal childbirth” [23] by not intervening obstetrically. It has been noted, however, that “taking time” and “*being with* […] rather than only *doing things*” as particular qualities of midwifery care practices have been “largely invisible” as they have not been “recorded […] monitored or accounted for” [25, 26]. Which interventions are prioritized in midwifery care practices—including what counts as an intervention; and whether, how, and when such interventions should be done, and with which effects—can only be investigated through empirical specification. Studying midwifery practices can also help to clarify and to improve midwifery‐specific concepts, such as “physiology” or “woman‐centered care”: What these concepts or ideals stand for can only be understood through addressing the realities of physiology‐in‐practice or woman‐centered‐care‐in‐practice in midwifery research.

It is my contention that this is not only a call to midwifery to articulate midwifery‐specific interventions or techniques in their own terms but also an invitation to reconsider midwifery‐specific notions of physiology and non‐intervention, and of woman‐centered care, empowerment, and choice. Seemingly unambiguous in theory, these normative concepts forming a midwifery ethos are not necessarily productive in practice where different “goods” are pursued and co‐exist with “bads” which do not align [27].

Our colleagues have framed ethical tensions that characterize everyday midwifery care practices as dilemmas. The routine necessity of balancing the benefits of promoting physiology by not intervening obstetrically “with the need to intervene appropriately when pathology arises in the complex, dynamic, highly individual situation of labour” would pose “a ‘wicked’ problem […]” in everyday midwifery care practices [23]. Decisions regarding whether, when, and how (not) to intervene “resist clear definitions, defy traditional analysis approaches, and refuse definitive resolution through predetermined solutions” [23].

Midwifery Care Studies build on Faye Thompson's suggestion that “midwifery mostly involves ‘everyday’ rather than crisis decision making” [28]. Against that backdrop, Midwifery Care Studies explore how exactly midwifery care practices engage with ethical and practical tensions in ways that *avoid* or *work around* framing women and fetuses as opposing individuals, as well as the impossible choices such oppositions may create. This approach draws on the concept of *tinkering*, as used in STS‐inspired studies of care, to illustrate how pregnancy, birth, and postpartum are contingent and complex events and processes. These processes require creative, responsive, and ongoing engagement in everyday midwifery care. This engagement may take the form of actively and perceptively (not) doing something or through an active‐passive supportive presence [3]. Before administering oxytocin, a midwife and a birthing person could engage in non‐medical strategies to speed up labor, such as encouraging movement and changing positions. A compromise for a birth giver refusing a medically advised cesarean could involve closely monitoring labor progression and fetal well‐being for a set period while providing support and encouragement, observing the progress of labor before reassessing the need for a cesarean.

## 3 Where We Stand II: A Methodological Torch for Midwifery

Committing to understanding midwifery care as radically relational [29], Midwifery Care Studies avoid distancing techniques, such as generalizing and juxtaposing, for example, midwifery and obstetrics. Biomedical nomenclatures, technologies of surveillance, and ideals of health form integral parts of daily midwifery care practices in many maternity care environments around the globe. Consequently, this approach aimed at investigating how and to what ends they are integrated, adapted, and transformed in everyday midwifery care practices of working‐with [30, 31, 32, 33].

The Midwifery Care Studies approach responds to the call of one of the protagonists of evidence‐based obstetrics, Canadian obstetrician Murray Enkin to step “beyond evidence” in order to articulate the “complex interrelationships” that characterize maternity care situations [34]. Midwifery Care Studies add to evidence‐based approaches by applying care‐specific modes of reflective intervention that do justice to the processual, contingent, and relational character of midwifery care practices. As suggested by Newnham and Rothman [1], Midwifery Care Studies attend to relational midwifery care techniques and embodied knowledge repertoires. Midwifery Care Studies conceive of working‐with as encompassing many different embodied and embodying practices, including to “sit quietly, [to] speak gentle encouraging words […], [to] prepare some drinks or [to] give a massage” and to use all senses “to observe a woman's wellbeing and labour progress” [25] as sets of active, consequential and highly skilled midwifery care techniques.

The approach commits to developing adequate conceptual and methodical toolkits to do so by adapting participatory and creative methods, such as those used in ethnographic or praxiographic research [33]. Similar to midwifery‐specific care techniques, these research methods require an attentive and reflective *working‐with* when collecting and analyzing empirical data.

Midwifery Care Studies share phenomenology's interest in understanding the material and social conditions of possibility for specific embodied subject positions to emerge and for the experiences they give rise to. In so doing, this approach avoids privileging neoliberal notions of choice and control that presume self‐contained disembodied subjects. Although these notions have been critically discussed within midwifery [24, 28, 35, 36, 37, 38], these discussions have not yet led to re‐examining midwifery‐specific concepts that often refer to such a logic of choice, for example, woman‐centered care or empowerment.

Extending the biomedical scope of (non‐)intervention concerning diagnosis and treatment, Midwifery Care Studies articulate the effects of situated modes of working‐with in midwifery care, shifting the focus beyond standardized health outcomes toward broader notions of a “good” life. What “good” pregnancies and “good” births might entail in midwifery care is essentially contested and is thus a political issue—and it needs to be addressed as such. What to do in midwifery care practices, the most important clinical question propelling evidence‐based interest in “what works”, can only be answered satisfactorily if the medical goals of saving life and restoring health are approached as questions rather than taken uncritically as givens [39].

## 4 Conclusion: Fostering Generative Research and Care Interventions

Midwifery care is marked by engaging in practical activities to make living through pregnancy, birth, and the months that follow “better”. How exactly to do “good” cannot, however, be decided beforehand, but is established in and through situated caring activities. It is a matter of ongoing creative and collective, embodied, and situated efforts and not merely the application of scientific knowledge and ethical principles. Taking the intricacies of midwifery care practices seriously, Midwifery Care Studies examine midwifery care practices as local dealings with specific life situations grappling with different value, knowledge, and technology repertoires. This includes specifying midwifery practices’ normative orientations, the kinds of “goods” midwifery practices and their participants live up to, the frictions they give rise to, and the ways they are dealt with in midwifery research.

In cultivating reflexive and transformative methodical tools, Midwifery Care Studies aim at providing the conceptual and analytical resources for fostering generative dealings with a multitude of specific lived realities of being pregnant and giving birth in midwifery research and practice. Through tinkering, midwives and the people they take care of engage in small, iterative adjustments in the course of unfolding situations, rather than rigidly adhering to standardized protocols. They focus on working through tensions in ways that are relational, adaptive, and creative. In summary, generative dealings in midwifery care are about actively and creatively engaging with the specificities of each situation to provide care that is responsive to the lived realities of pregnancy and birth. This approach moves beyond rigid frameworks, emphasizing the importance of flexibility, creativity, and attentiveness in everyday midwifery care practices.

## Conflicts of Interest

The author declares no conflicts of interest.

## Data Availability

Data sharing not applicable to this article as no datasets were generated or analysed during the current study.
